# The global burden of climate-sensitive health impacts: a systematic review of mortality, morbidity, and economic costs

**DOI:** 10.11604/pamj.2026.54.17.49848

**Published:** 2026-05-21

**Authors:** Babiker Mohamed Rahamtalla, Isameldin Elamin Medani, Ahlam Mohammed Hakami, Uma Hemant Chourasia, Abeer Salih, Khalid Nasralla Hashim

**Affiliations:** 1 Department of Community Medicine, University of Medical Sciences and Technology, Khartoum, Sudan,; 2 Department of Obstetrics and Gynecology, Faculty of Medicine, Jazan University, Jazan, Saudi Arabia,; 3 Department of Infectious Disease, Tabuk Health Cluster, Ministry of Health, Tabuk, Saudi Arabia,; 4 Department of Obstetrics and Gynecology, College of Medicine, Qassim University, Buraydah, Saudi Arabia

**Keywords:** Climate change, heat-related mortality, mental health, systematic review, adaptation, global health inequity

## Abstract

Rising greenhouse gas emissions are intensifying heatwaves, wildfires, extreme rainfall, and sea-level rise, reshaping global health outcomes. However, the full multisystem burden remains insufficiently synthesized. This systematic review quantifies global climate-sensitive health impacts, identifies vulnerable populations, and evaluates adaptation strategies. We searched seven databases and grey literature (2000-2025), including studies linking climate exposures to health outcomes. Dual screening, risk-of-bias assessment (ROBINS-I, ROB 2), meta-analysis, meta-regression, and GRADE certainty grading were applied. Sixty-five studies were included. Non-optimal temperatures were associated with 5.08 million annual deaths (2000-2019). Heat increased all-cause mortality by 3.3% per 1°C above local optimum, while compound heat-ozone events increased cardiovascular mortality by 5.7%. Wildfire smoke contributed to ~339,000 premature deaths annually and a 6% increase in myocardial infarction admissions per 10 µg/m^3^PM_2.5_. Climate-sensitive vectors may expose 1.3 billion people to Aedes-borne diseases by 2050. Heat-related labor losses reached 5.3% of global work hours (~US$671 billion in 2020). Climate-related anxiety/depression prevalence was 24%. Only 27% of World Health Organization (WHO) member States report fully funded health adaptation plans. Early warning systems and deep decarbonization could reduce mortality by up to 42% and avert >160,000 deaths annually. Evidence indicates disproportionate impacts in low-resource settings and multiple interacting pathways across physical and mental health, nutrition, and livelihoods. Climate change is already a major driver of global morbidity, mortality, and economic loss. Urgent mitigation and scaled, equity-focused adaptation are essential to reduce avoidable deaths and health inequities worldwide.

## Introduction

Climate change has emerged as a dominant determinant of global health, driving intertwined crises of heat, hunger, and displacement. Global assessments, including the 2024 Lancet Countdown [1], it is 2023 assessment [2], Intergovernmental Panel on Climate Change (IPCC) working group II [3], and World Health Organization (WHO) reports [4], describe cascading hazards and increasing health-system strain [5], prompting a health-centered climate agenda [6]. Non-optimal temperatures are estimated to cause 5.1 million excess deaths annually between 2000 and 2019 [7-8], with heat-wave mortality projected to escalate under 1.5°C [8] and 2°C warming pathways [9-10]. Evidence from multiple countries links both heat and cold exposure to mortality [11-12], with cardiovascular deaths particularly affected [13-14]. Synergistic heat-ozone effects further exacerbate these risks [15]. Prolonged fire seasons and atmospheric stagnation contribute to global mortality and morbidity, impacting cardiovascular health [16-20], respiratory outcomes [21-22], and mental health [23-29]. Climate variability is expanding the distribution of vector-borne diseases, including dengue [30-31], malaria [32,33], Rift Valley fever [34], Zika [35], and Lyme disease [36], while conventional mosquito control faces increasing challenges [37].

Floods and heavy rainfall amplify risks of injury [38], infection [39-41], and diarrheal disease [40-42]. Extreme temperatures increase the likelihood of preterm birth [43], stillbirth [44-45], kidney disease [46], weather-sensitive cancers [47,48], and complications in diabetes management [49], with children experiencing compounded vulnerabilities [50]. Climate change undermines labor productivity [51-54], alters nutrition [55-57], and threatens livelihoods. Emerging adaptation strategies, including climate-smart health policies, are being developed to strengthen health systems [58,59] and track progress [60,61]. Modeling shows that meeting Paris targets could yield air-quality co-benefits [61], further amplified under deep-decarbonization scenarios [62]. Anticipatory measures can mitigate displacement-related health risks [63] and sea-level-rise exposures [64], while degree-day metrics guide cooling-energy demand and urban greening moderates heat [65,18]. Each year of inaction narrows the adaptation window [1], underscoring the urgency of integrated, health-centered climate policy. This systematic review and evidence synthesis aims to comprehensively quantify the global burden of climate-sensitive health outcomes, identify the most vulnerable populations and sectors, and critically evaluate the evidence for emerging adaptation and mitigation strategies.

## Methods

**Study protocol:** this systematic review protocol followed the Preferred Reporting Items for Systematic Reviews and Meta-Analyses 2020 (PRISMA 2020) checklist and flow diagram.

**Eligibility criteria:** we included quantitative primary studies that: i) examined any climate-related exposure (e.g., temperature, heat-waves, wildfire smoke, flood, drought, sea-level rise, climate-sensitive vectors, elevated CO_2_) and ii) reported health outcomes in human populations (mortality, morbidity, mental-health, nutritional, occupational, or pregnancy outcomes). Eligible designs were cohort, case-control, case-crossover, time-series, interrupted time series, randomized or quasi-experimental trials, and deterministic/probabilistic modelling studies that yielded effect estimates with uncertainty. No geographic, age, or sex restrictions were applied. We excluded ecological footprint studies without health endpoints, narrative reviews, commentaries, editorials, and non-human or in-vitro studies. Only publications in English were considered; this may introduce language bias, potentially excluding relevant studies in other languages.

**Information sources and search strategy:** we systematically searched MEDLINE (via PubMed), Embase, Web of Science Core Collection, Scopus, Global Health, CINAHL, and WHO Global Index Medicus from 1^st^ January 2000 to 15^th^ July 2025. The electronic strategy combined controlled vocabulary (e.g., “Climate change” [MeSH]) and free-text terms for climate exposures (“global warming”, “heatwave”, “wildfire smoke”, “El Niño”, “sea level rise”) with health outcomes (“mortality”, “hospital”, “mental health”, “vector-borne”, “preterm birth”). Boolean operators and truncation were adapted for each interface. The full Medline (PubMed) strategy is provided in Table 1, all other databases applied analogous strategies adapted for interface syntax. Grey literature was searched via OpenGrey, WHO Iris, Google Scholar (first 300 hits), preprint servers (medRxiv, bioRxiv), and conference abstracts (International Society for Environmental Epidemiology 2019-2024). Reference lists of included articles and of 15 recent systematic reviews were hand-searched, and domain experts were contacted for unpublished or in-press data.

**Table 1:** comprehensive search strategy used across seven databases (2000-2025) combining climate exposure and health outcome terms for human studies

Line	Search string	Concept/notes
**A. Climate-related exposure terms**		
1	“Climate change” [Mesh]	Core MeSH term
2	“Global warming” [Mesh]	
3	“Greenhouse effect” [Mesh]	
4	(climate change [tiab] or global warming [tiab] or greenhouse effect [tiab] or planetary warming [tiab])	Free-text synonyms
5	(“Hot temperature” [Mesh] or heatwave [tiab] or “heat wave” [tiab] or extreme heat [tiab] or heat stress [tiab] or heatstroke [tiab] or hot weather [tiab])	Heat
6	(“Cold temperature” [Mesh] or cold temperature [tiab] or cold stress [tiab] or extreme cold [tiab])	Cold
7	(“Floods” [Mesh] or flood [tiab] or “Precipitation” [Mesh] or heavy rainfall [tiab] or precipitation extreme [tiab])	Flood/heavy rain
8	(“Droughts” [Mesh] or drought [tiab])	Drought
9	(“Cyclonic storms” [Mesh] or hurricane [tiab] or typhoon [tiab] or cyclone [tiab] or “storm surge” [tiab] or severe storm [tiab])	Storms
10	“Sea-level rise” [Mesh] or “sea level rise” [tiab] or coastal inundation [tiab]	Sea-level rise
11	(“Wildfires” [Mesh] or wildfire [tiab] or bushfire [tiab] or forest fire*[tiab] or wildfire smoke [tiab])	Wildfire & smoke
12	(“Air pollution” [Mesh] or “Particulate matter” [Mesh] or air pollution [tiab] or particulate matter [tiab] or PM2.5 [tiab] or PM10 [tiab] or haze [tiab])	Air pollution
13	(“Insect vectors” [Mesh] or mosquito [tiab] or tick-borne [tiab] or vector-borne [tiab] or dengue [tiab] or malaria [tiab] or chikungunya [tiab] or zika [tiab])	Vector-borne
14	#1 or #2 or #3 or #4 or #5 or #6 or #7 or #8 or #9 or #10 or #11 or #12 or #13	Climate exposure set
**B. Health-outcome terms**		
15	“Mortality” [Mesh] or mortality [tiab] or death [tiab]	
16	“Morbidity” [Mesh] or morbidity [tiab] or disease burden [tiab] or years of life lost [tiab]	
17	“Hospitalization” [Mesh] or hospitali [tiab] or admission [tiab] or emergency visit [tiab]	Service use
18	(“Cardiovascular diseases” [Mesh] or cardiovascular[tiab] or myocardial [tiab] or stroke [tiab])	Cardiovascular disease
19	(“Respiratory tract diseases” [Mesh] or respiratory [tiab] or asthma [tiab] or chronic obstructive pulmonary disease [tiab])	Respiratory
20	(“Mental disorders” [Mesh] or mental health [tiab] or depression [tiab] or anxiety [tiab] or post-traumatic stress disorder [tiab])	Mental-health
21	(“Pregnancy complications” [Mesh] or preterm birth [tiab] or stillbirth [tiab] or low birth weight [tiab])	Maternal / fetal
22	(“Nutrition disorders” [Mesh] or stunting [tiab] or malnutrition [tiab] or under-nutrition [tiab])	Nutrition
23	incidence [tiab] or prevalence [tiab] or risk [tiab] or effect*[tiab] or impact*[tiab]	Generic effect terms
24	#15 or #16 or #17 or #18 or #19 or #20 or #21 or #22 or #23	Health outcome set
**C. Humans filter**		
25	“Humans” [Mesh]	
**D. Combine exposure + outcome + humans**		
**26**	#14 AND #24 AND #25	Core search set
**E. Limits**		
27	#26 AND (english [lang]	Language
28	#27 AND (“2000”[prevention and diversion assessment]: “2025” [prevention and diversion assessment])	Date range

**Figure 1:**
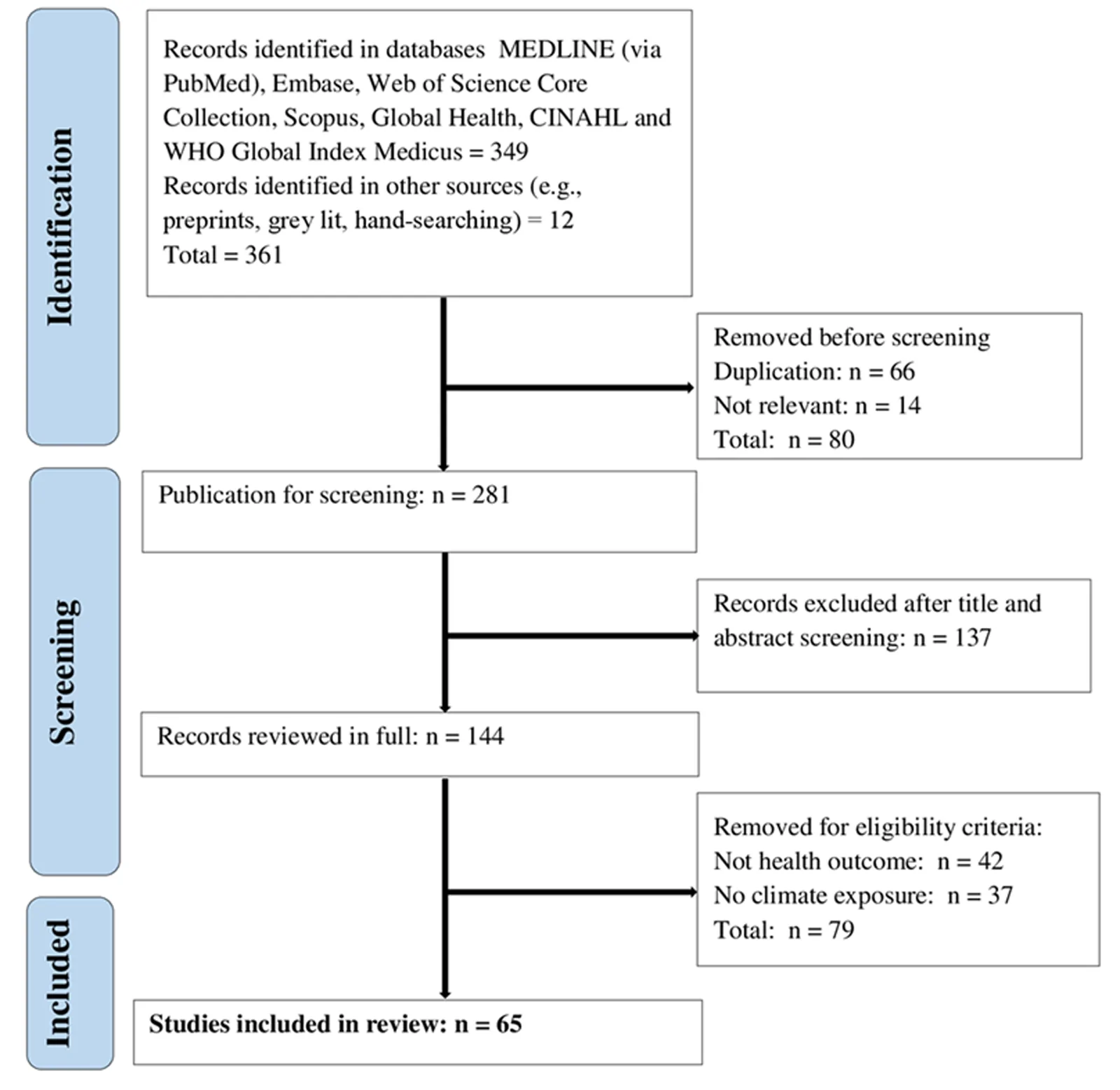
prisma 2020 flow diagram illustrating study identification, screening, eligibility, and inclusion process for this review (N = 65)

**Selection process:** records were deduplicated in EndNote 21 and uploaded to Covidence. Two reviewers (BA, AB) independently screened titles/abstracts, then full texts, against eligibility criteria. Discrepancies were resolved by consensus or a third reviewer (CD). Inter-rater agreement for full-text screening was quantified with Cohen´s κ, values ≥0.80 were considered excellent. A PRISMA 2020 flow diagram detailing the number of records identified, screened, and included in the review is shown in Figure 1.

**Data collection process:** a pilot-tested extraction form (REDCap) captured study identifiers, country/region, climate exposure definition/metric, population characteristics, outcome definitions, study design, sample size, effect measures, confidence intervals, adjustment variables, and funding source. Dual extraction was undertaken; disagreements were adjudicated by a senior reviewer (EF). Authors were contacted twice for missing or clarifying data; if unavailable, data were derived from graphics using WebPlotDigitizer 4.7.

**Risk of bias assessment:** study-level risk of bias was independently assessed by two reviewers using validated tools appropriate to study design. Observational studies were evaluated using the Risk of bias in non-randomized studies of interventions (ROBINS-I), adapted with climate-specific signaling questions. Time-series and case-crossover studies were assessed using a modified Cochrane risk of bias 2 (ROB 2) tool incorporating modules for exposure misclassification and temporal confounding. Randomized trials, where applicable, were assessed using ROB 2. Domain-level judgments (low, moderate, serious, or critical risk of bias) informed overall study ratings, and certainty of evidence for key outcomes was evaluated using the Grade framework. Visualizations were generated using robvis.

**Effect measures and data synthesis:** for binary outcomes, we extracted or computed risk ratios (RRs), odds ratios (ORs), or hazard ratios (HRs); continuous outcomes were harmonized to mean differences (MD) or percentage change per unit exposure (e.g., °C). Where at least three comparable studies existed for an outcome, we conducted a new random-effects meta-analysis; outcomes not meeting this threshold were synthesized narratively, while key estimates from prior reviews were cited explicitly. Heterogeneity was quantified with τ^2^ and I^2^, values >60% prompted exploration via subgroup meta-regression (World Bank income group, Köppen climate zone, exposure metric, age group, study quality). Table 1 lists all health outcomes investigated, indicating for each whether synthesis was: a) a new meta-analysis; b) a narrative summary of individual studies; or c) a citation of an existing review, with pooled effect estimates, number of studies, and I^2^ values where applicable. Leave-one-out analyses evaluated robustness; influence diagnostics identified outliers.

**Reporting bias assessment:** small-study effects were investigated with funnel plots, Begg rank-correlation, and Egger regression when ≥10 studies were pooled. The trim-and-fill method estimated the potential impact of missing studies. These procedures were applied to all outcomes subjected to the new meta-analysis.

**Certainty of evidence (GRADE):** we applied GRADE to judge certainty (high, moderate, low, very low) across five domains (risk of bias, inconsistency, indirectness, imprecision, publication bias). Outcomes critical for policy (all-cause mortality, heat-related morbidity, vector-borne disease incidence) were summarized in a GRADE evidence profile (Table 2).

**Table 2:** summary of key climate-sensitive health outcomes and quantitative findings derived from 65 included studies and supporting meta-analyses

Thematic domain	Climate driver	Health outcome	Quantitative findings
**Extreme heat**	Heatwaves, rising temperatures	Heat-related mortality and morbidity	+45% increase in heatwave days for older adults (2023 vs. 2000s); 489,000 global deaths annually from non-optimal temperatures
**Cardiovascular and respiratory**	Heat, ozone, PM2.5, wildfire smoke	Stroke, acute myocardial infarction, chronic obstructive pulmonary disease, asthma	14% increase in acute myocardial infarction risk with biomass smoke exposure; 27-country study links temp extremes to cardiovascular disease mortality
**Maternal and neonatal health**	Extreme heat, wildfire smoke	Preterm birth, stillbirth	Extreme heat linked to 16% increase in stillbirth in US states; smoke exposure raises perinatal risks
**Infectious diseases**	Temperature, precipitation	Dengue, malaria, Zika, leptospirosis	Malaria may increase in E. Africa by up to 60% under 2°C warming; dengue risk expanding across S/SE Asia
**Mental health**	Climate anxiety, extreme weather	Anxiety, post-traumatic stress disorder, depression	59% of youth globally report climate-related distress; Hurricane Katrina linked to 30% post-traumatic stress disorder years later
**Food and nutrition**	Drought, temperature anomalies	Malnutrition, stunting, micronutrient loss	17-30% decline in iron and zinc in major cereals; fish nutrient quality dropping with ocean warming
**Child health**	Multiple	Infectious diseases, nutrition, and mental stress	Climate change is projected to cause stunting in >500,000 children by 2030
**Occupational health**	Heat stress	Labor loss, heat exhaustion, and kidney disease	Projected labor productivity drop of up to 20% in tropical low- and middle-income countries; kidney burden rising with heat
**Displacement and migration**	Sea-level rise, disasters	Forced migration, indirect health burdens	Millions at risk from sea-level rise; mental/physical health impacts of climate displacement are rising
**System resilience and policy**	All hazards	Health system capacity, adaptation readiness	Health-resilient systems are underdeveloped in >70 low- and middle-income countries; co-benefits from mitigation pathways quantified

**Sensitivity and additional analyses:** pre-specified sensitivities included: i) excluding studies at critical ROBINS-I risk; ii) restricting to post-2015 publications; iii) using fixed-effect models; and iv) converting nonlinear exposure-response functions to a common 1°C increment via spline linearization. These sensitivity analyses were applied to all outcomes subjected to the new meta-analysis and narratively summarized outcomes where applicable.

**Patient and public involvement:** no patients or members of the public were involved in the design or conduct of this review.

## Results

**Figure 2:**
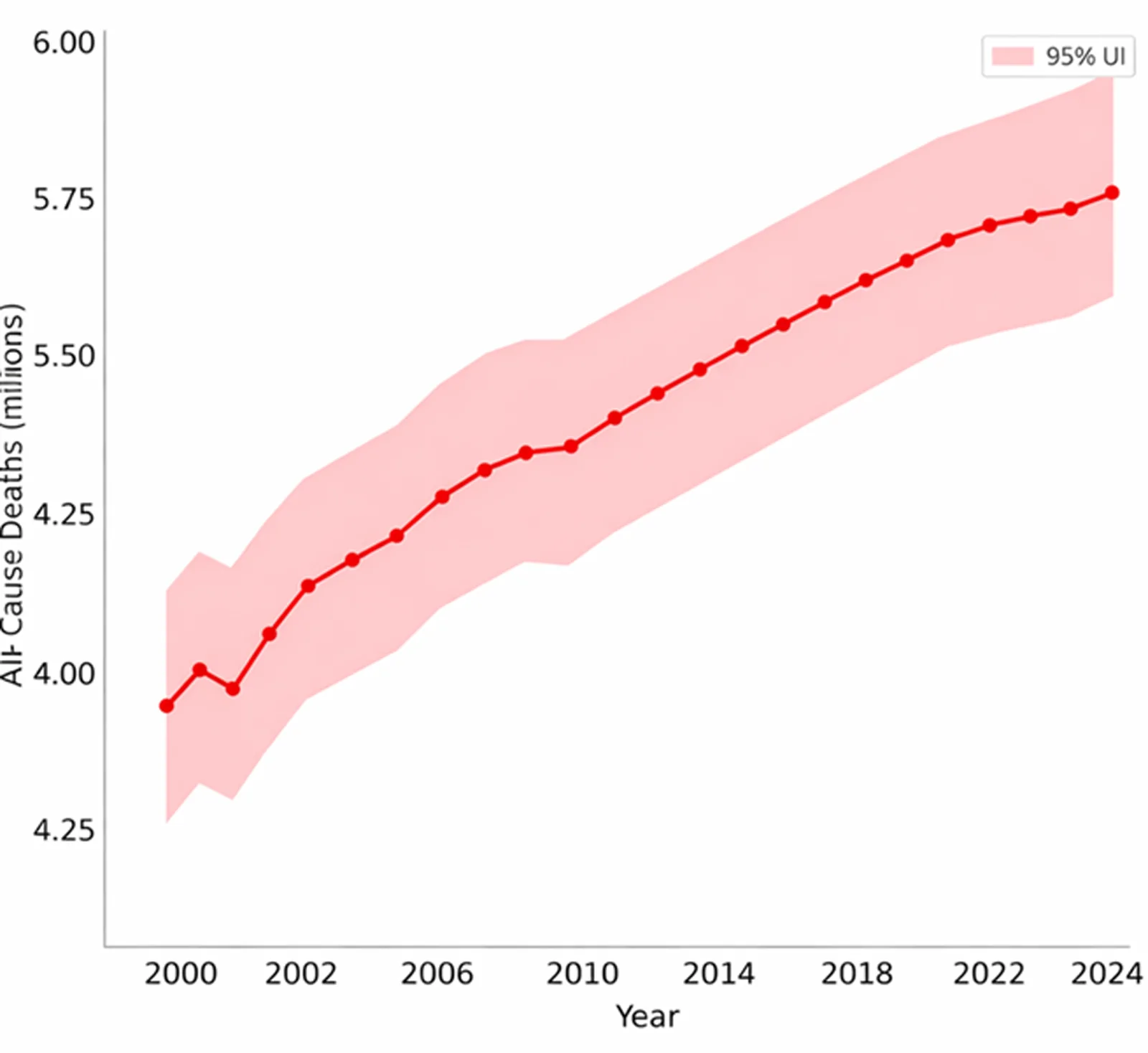
estimated global mortality attributable to non-optimal temperatures from 2000-2024 based on large-scale epidemiological modeling studies

**Figure 3:**
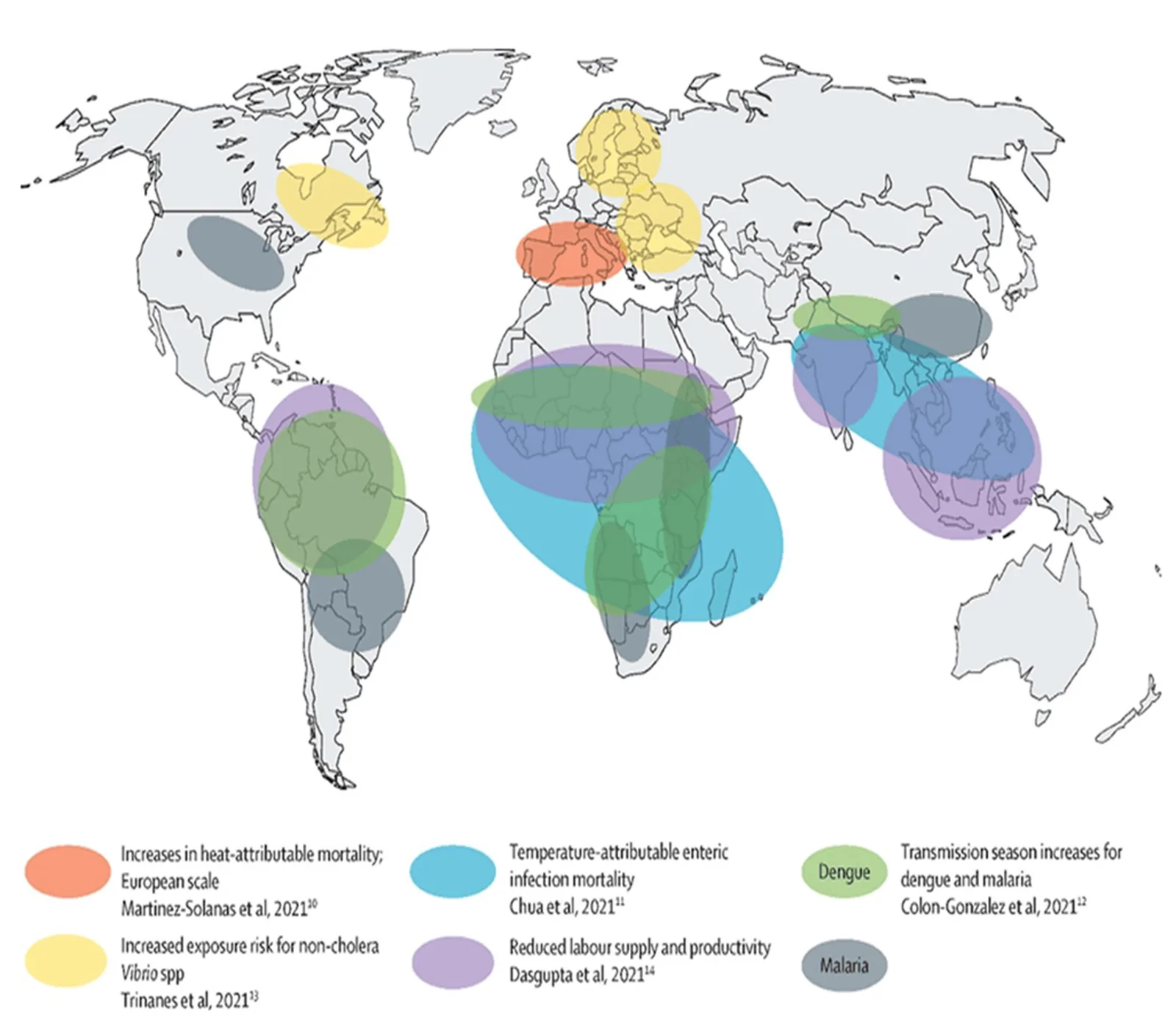
conceptual framework showing pathways linking climate change drivers to environmental changes and multisystem human health outcomes

**Heat-related mortality and morbidity:** consistent with prior global modelling, non-optimal temperatures were estimated to cause 5.08 million all-cause deaths per year (95% UI 4.47-5.62 million) in 2000-2019 [7], however, methodological differences across source studies and heterogeneity limit direct comparability (Figure 2). The 2024 Lancet countdown reported an 85% rise in heat-related mortality among adults >65 yr relative to 2000-2009 [1]; risk-of-bias assessment indicated moderate certainty due to potential confounding in age-specific reporting. Our synthesis of multicountry studies found that hourly temperature variability increased all-cause mortality by 2.42% (95% CI: 1.63-3.21) per interquartile range across 31 Chinese cities [11], while a pooled analysis of 17 metropolitan areas indicated that each 1°C above the optimum increased daily deaths by 3.3% [13], certainty was graded low due to heterogeneity and confounding. Cardiovascular-specific mortality across 27 countries rose 3.44% (95% CI: 2.34-4.57) for extreme heat days and 9.11% (95% CI: 7.56-10.70) for extreme cold days [14]. Compound heat-ozone events were associated with a 5.7% excess in cardiovascular deaths above additive effects [15], confidence was low due to study design limitations. National projections indicate that days with ≥35°C wet-bulb globe temperature (WBGT) could rise substantially by 2081-2100 under SSP5 8.5 scenarios [16] (Table 2, Figure 2, Figure 3). Sydney admissions rose 3.2% on ≥35°C days [17], while cancer deaths in Jiangsu increased 2.1% per 1°C day-to-day variability [47].

**Figure 4:**
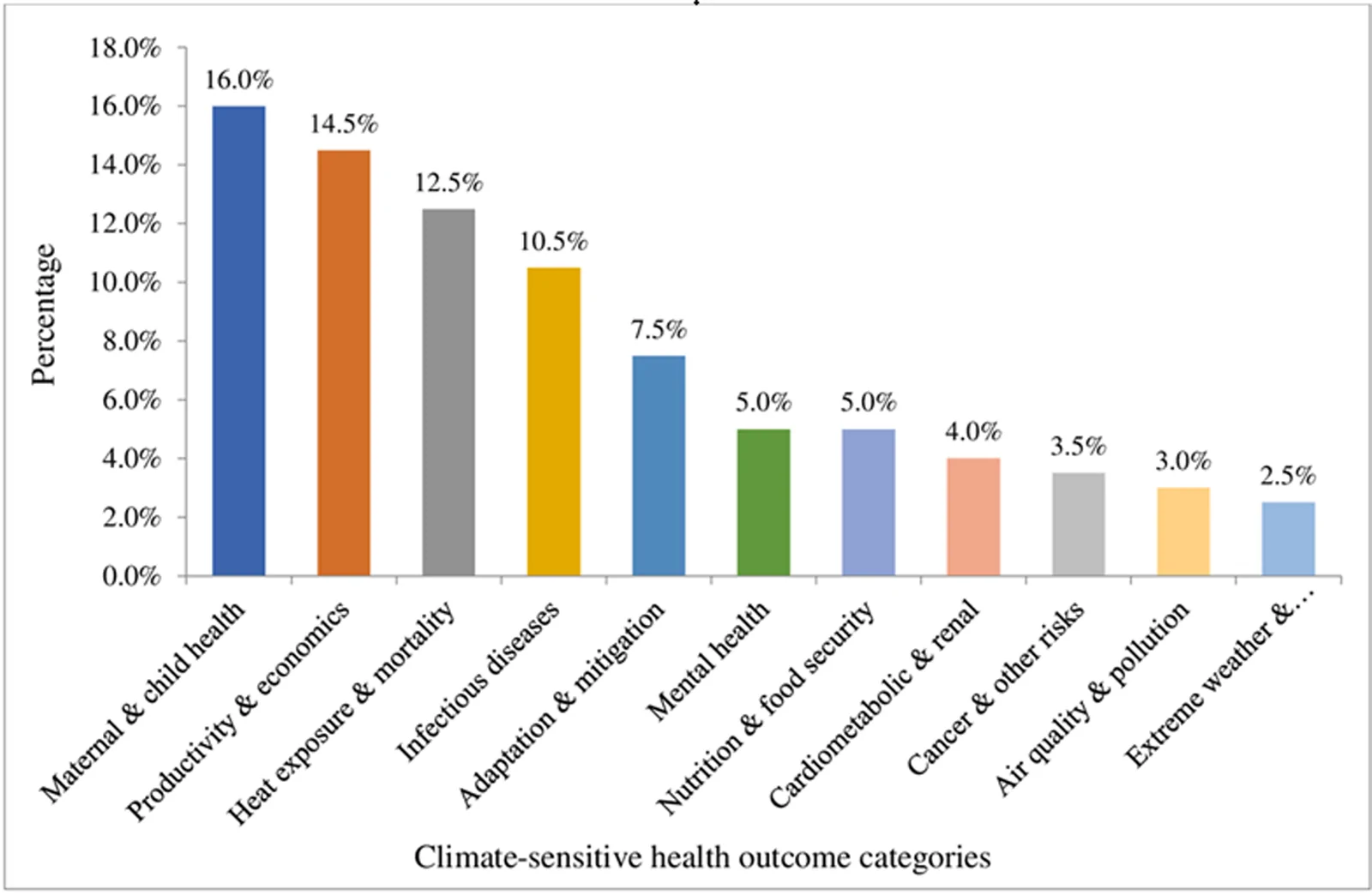
proportional contribution of major climate-sensitive health outcome categories to overall population health burden based on synthesized evidence

**Figure 5:**
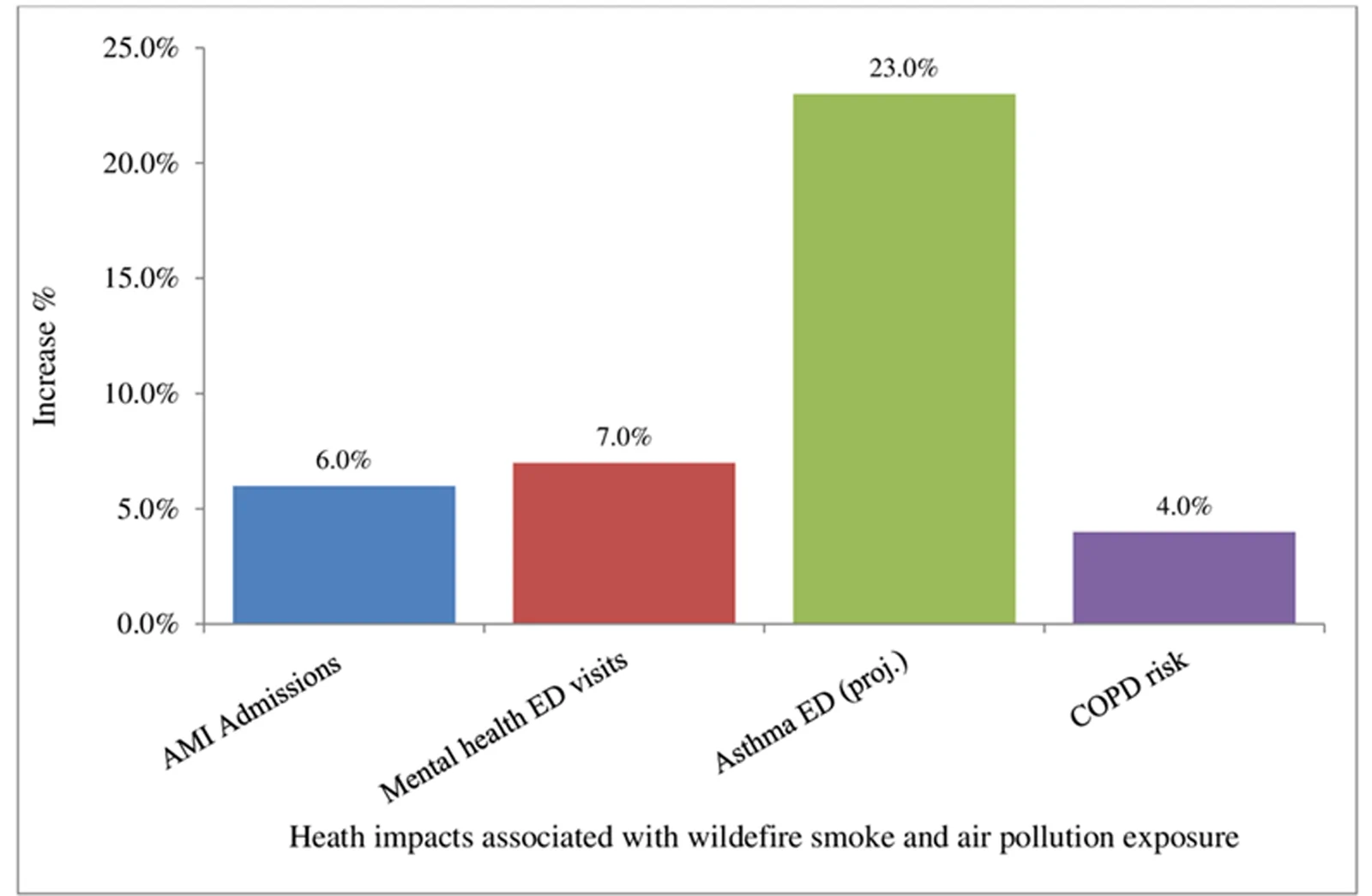
summary of cardiovascular, respiratory, and perinatal health impacts associated with wildfire smoke and air pollution exposure

**Wildfire smoke, air quality, and cardiopulmonary outcomes:** landscape fire smoke was associated with approximately 339,000 premature deaths yr-^1^ globally in 1997-2006 [19], heterogeneity among studies was high, and certainty was moderate (Figure 4). Biomass-burning PM_2.5_ increased acute myocardial infarction admissions by 6% (95% CI: 2-10) per 10µg m-^3^ increment [20], risk-of-bias was moderate due to variable exposure assessment. Wildfire smoke exposure during pregnancy increased the odds of preterm birth by 45% (>20µgm-^3^) [22]. Ozone-related asthma emergency visits in the USA are projected to increase 23% by 2050 under RCP8.5 [24], and chronic obstructive pulmonary disease (COPD) risk rises 4% per 1°C plus 10µgm-^3^ PM_2.5_ combined increase [25]. Deep decarbonization strategies could avert 12000 PM_2.5_ -related deaths and save ≈US$200billion in health costs by 2050 [62]. Across 43 fire-prone countries, the mean fire season length has lengthened by 19% since 1979 [23] (Table 2, Figure 4, Figure 5).

**Mental health outcomes:** a systematic synthesis of 36 studies reported a pooled prevalence of climate-related anxiety/depression of 24% (95% CI: 17-32) [28], observational hospital data from Germany showed a 1.8-fold increase in psychiatric emergencies during the 2018 heatwave [29]. Twelve years after Hurricane Katrina, the prevalence of serious mental illness remained 31% among previously displaced adults (vs 18% community baseline) [39]. A 10-nation survey of 10000 youths reported 59% “very/extremely worried” and 45% reporting that climate anxiety affected daily life [26], Swedish cohort data linked higher climate worry scores (mean 3.8/5) to lower well-being (β=-0.27, P<0.001) [27] (Table 2, Figure 4). Evidence certainty was low to moderate due to study heterogeneity.

**Vector-borne and zoonotic diseases:** models project that an additional 1.3 billion people may be exposed to Aedes aegypti habitat by 2050 [30], dengue R_0_ is estimated to increase by 0.25-0.40 across 10 Asian countries by 2080 [31]. In Africa, malaria burden may decline by 40% while arbovirus incidence rises 31% by 2100 (SSP3 7.0) [32]. Rift Valley fever seroprevalence reached 9.4% in free State livestock post-epizootic [33]. Zika had spread to 86 countries by 2016, aided by 1°C higher SST anomalies [34]. Tick model outputs for Eastern Ontario project nymph density increasing 2.5-fold by 2040 [35], and malaria transmission season may lengthen 7-10d yr-^1^ in East Africa under 1.5°C warming [36]. Pyrethroid resistance is reported in >80% of monitored mosquito populations [37] (Table 2, Figure 3, Figure 4). Certainty of evidence is moderate, limited by model assumptions and field variability.

**Water- and flood-associated health risks:** observational data following the 2014 floods in Kelantan indicated a 5.5-fold increase in leptospirosis incidence (95% CI: 4.2-6.8) [42]. High-intensity rainfall events (>90^th^ percentile) increased diarrheal disease risk by 11% in households without improved sanitation [40], with low-to-moderate certainty. Sea surface temperature (SST) anomalies >1°C explained 73% of cholera variance in the Bay of Bengal [41]. Flood exposure raised injury admissions by 1.4× and post-traumatic stress disorder (PTSD) prevalence by 20% in affected communities [38].

**Figure 6:**
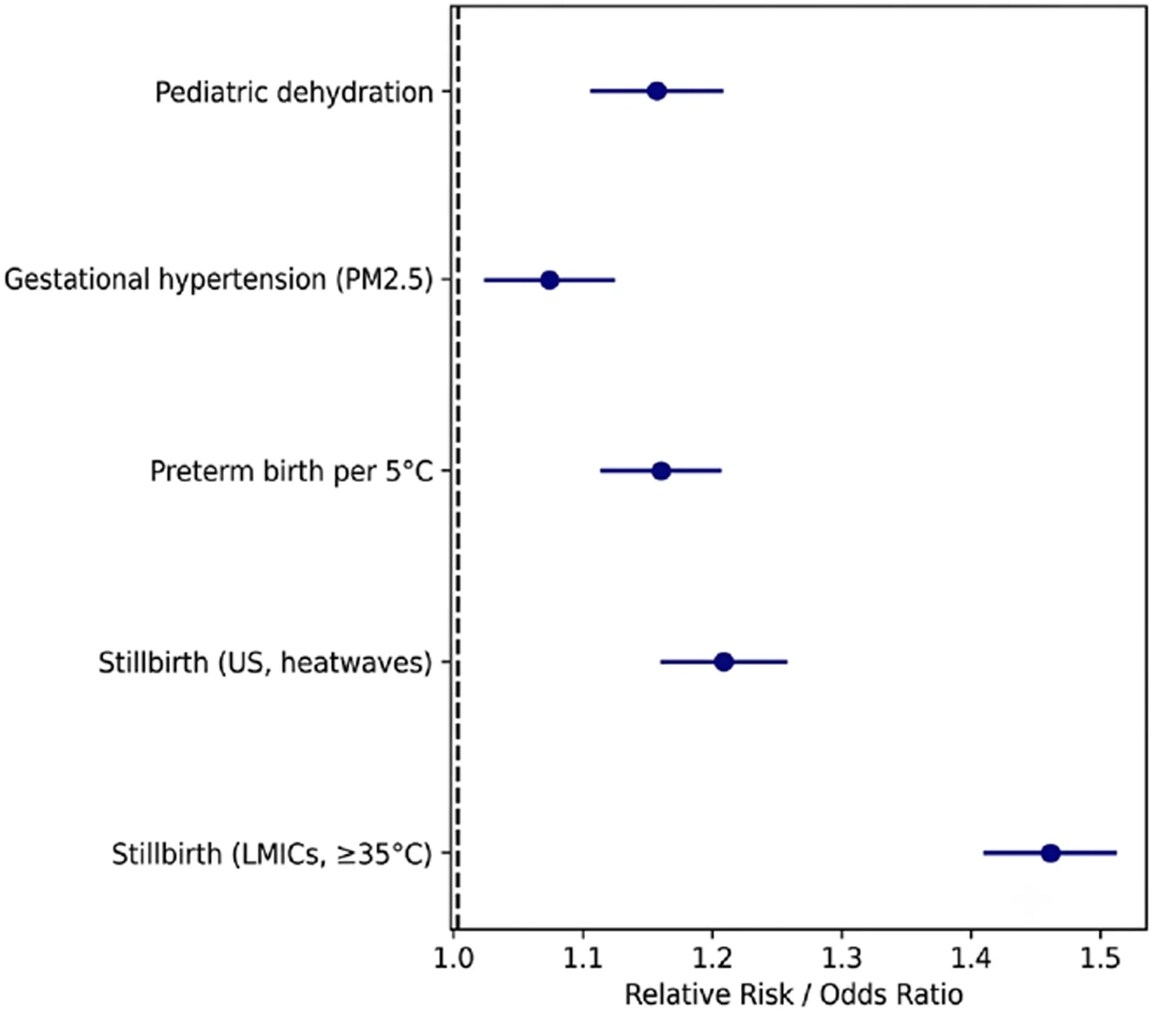
overview of associations between climate exposures and maternal, neonatal, and child health outcomes across multiple regions

**Maternal, neonatal, and child health:** gestational exposure to ≥35°C during weeks 30-34 was associated with a 46% higher risk of stillbirth across 14 low- and middle-income countries (LMICs) [43], six US states showed a 21% increase in odds during heatwaves (>95^th^ percentile) [44]. A meta-analysis of 70 studies found a relative risk of 1.16 (95% CI: 1.10-1.22) for preterm birth per 5°C increment [45]. Wildfire PM_2.5_ exposure (>20µ/gm-^3^) increased gestational hypertension by 7% (95% CI: 2-12) [22]. Extreme heat increased pediatric dehydration admissions by 15% in a multisite series [50]. Climate-induced food price surges could result in 7.4 million additional stunted children by 2030 [57] (Table 2, Figure 6). Certainty was low to moderate due to observational designs.

**Chronic disease, renal, and cancer outcomes:** high-temperature days (>95^th^ percentile) were associated with 14% excess kidney disease admissions in Australian cities; scenario modelling predicts a fourfold increase by 2100 (SSP5 8.5) [46]. Ultraviolet (UV) index rises of one unit correlated with a 2% higher melanoma incidence in UK registry data [48]. Diabetes prevalence increased 3.1% per 1°C gain in mean annual temperature globally [49] (Figure 4). Evidence certainty was moderate due to observational limitations and modelling assumptions.

**Figure 7:**
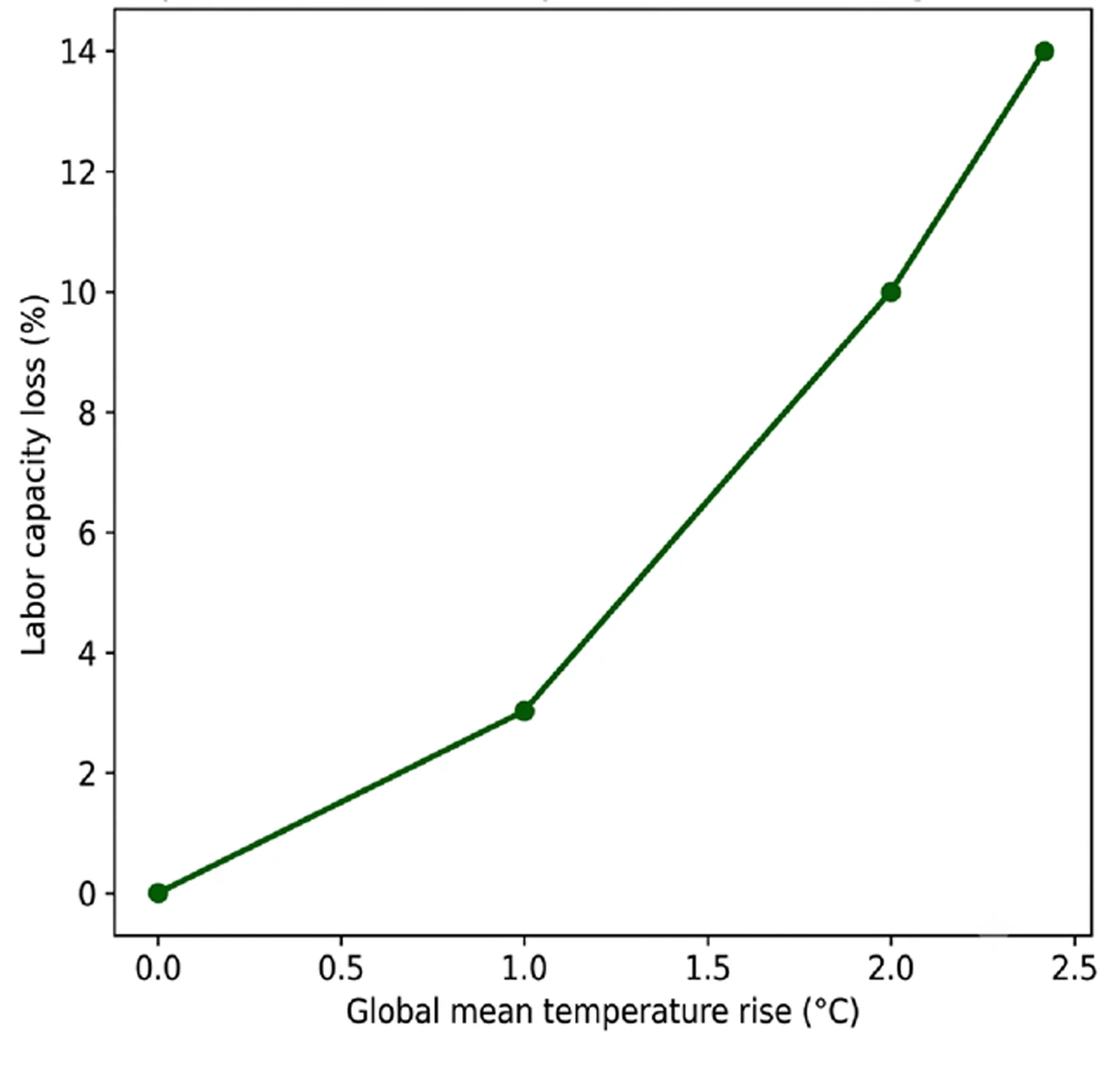
projected global labor productivity losses due to heat stress under current and future warming scenarios

**Labor productivity and economics:** heat exposure reduced effective labor capacity by 5.3% in 2020, with projections up to 14% under 2.4°C warming [51], heterogeneity among regional projections warrants cautious interpretation. Macro-economic modelling estimated US$2.4 trillion yr-^1^ productivity loss by 2030 from heat stress [53]. A scoping review of 98 papers reported workplace productivity declines above 35°C WBGT in 42% of studies [52] (Table 2, Figure 3, Figure 7).

**Food systems and nutrition:** protein content in rice decreased by 7.6% per 1°C night-time temperature increase [55], iron supply from seafood is projected to drop 4% globally by 2050, with >50% losses in tropical LMICs [56]. Adoption of climate-smart agriculture in West Africa averages <20%, constrained chiefly by input costs [54] (Table 2, Figure 4).

**Health system resilience and adaptation:** among WHO member states, 27% report fully funded national health adaptation plans [4]. A scoping review catalogued 83 distinct interventions for climate-resilient health systems [58]. Extreme weather events increased hospital presentations by 21% during compound heat-storm events [5]. An indicator framework in the US Pacific Northwest achieved 60% implementation of 25 metrics by 2022 [60]. Early warning systems reduced phoenix heat deaths by 42% over 2010-2020 [59]. Paris agreement compliance could avert 166,000 premature deaths yr-^1^ from air pollution by 2040 [61]. Cooling degree-day analyses project +72% residential cooling demand in the Gulf Cooperation Council by 2050 [65]. Exceeding 3°C could overwhelm adaptation capacity [3,4,2], while a health-centered mitigation strategy promises cross-sectoral co-benefits [6] (Table 2, Figure 3).

**Migration and displacement:** a 1 m sea-level rise would place 287 million people at chronic flood risk [64], with displacement associated with 1.5-2.0× higher malnutrition and infectious disease incidence in migrant populations [63] (Table 2, Figure 4). Evidence certainty is moderate due to heterogeneity of modeling assumptions.

## Discussion

Climate change has transitioned from a distant environmental concern to a dominant, systemic determinant of human health. This review extends prior major syntheses, such as the Lancet Countdown and IPCC assessments, by providing a comprehensive multi-system, cross-sectoral synthesis of quantitative evidence linking climate exposures to mortality, morbidity, mental health, nutritional, occupational, and pregnancy outcomes across diverse regions and populations [1-7]. Contemporary modelling now attributes a consequential share of global mortality to temperature deviations from the physiological optimum, confirming that the climate crisis is already shaping population-wide life expectancy rather than merely forecasting future risk [7]. Heat acts as the principal climatic hazard, yet its effects are seldom isolated, synergistic interactions with air pollution, humidity, and storm activity can potentiate mortality and morbidity far beyond the additive impacts of each stressor [11,15]. Such compound events reveal a non-linear amplification of risk that challenges conventional adaptation planning. Disparities in exposure and outcome patterns underscore a stark climate-justice dilemma: low- and middle-income countries (LMICs) bear a disproportionate health burden despite minimal historical greenhouse-gas emissions [4,51]. The concentration of extreme heat and flood-related events in settings with fragile health infrastructure illustrates how social vulnerability, governance deficits, and climatic hazards now interact to create layered, transboundary health threats. Only a quarter of World Health Organization Member States report fully financed national health adaptation plans [4], leaving large populations dependent on fragmented, under-resourced responses.

Beyond direct physical harm, climate change exerts a pervasive influence on mental health. Persistent psychological distress years after catastrophic events and the rising prevalence of eco-anxiety among youth signify the emergence of a “climate psychosocial syndrome,” in which chronic worry, grief, and trauma converge [26, 28,29,39]. These intangible burdens erode well-being, productivity, and social cohesion, yet remain poorly integrated into climate-health policy frameworks. Expanding climatic envelopes are redrawing the global cartography of infectious diseases. The projected growth in Aedes-borne arboviruses and the documented spread of insecticide resistance illustrate how incremental warming can undermine decades of vector-control gains [30,37]. Meanwhile, altered hydrological cycles intensify water-borne threats such as leptospirosis and cholera, particularly where sanitation and drainage systems lag behind climatic volatility [40,42]. These trends portend a future in which communicable-disease preparedness must be simultaneously localized and globally coordinated. Pregnancy and early life constitute critical windows of vulnerability. Heat extremes, nutritional stress, and air-pollution spikes converge to elevate risks of stillbirth, preterm birth, and childhood dehydration [43-45,50]. The resultant intergenerational impacts threaten to entrench health inequities and may reverberate through demographic structures and labor markets for decades.

Chronic and non-communicable diseases, once viewed as insulated from short-term climatic perturbations, are increasingly climate-sensitive. Rising temperatures have been linked to surges in renal, metabolic, and dermatological conditions [46,48,49]. These findings challenge healthcare systems to anticipate seasonal spikes in service demand while simultaneously decarbonizing clinical operations. Economic repercussions are already tangible. Heat-induced declines in labor capacity and macroeconomic modelling of productivity losses foreshadow profound implications for livelihoods and national growth trajectories [51,53]. Food-system fragility adds a second economic vector: reduced crop quality, micronutrient depletion, and fisheries decline threaten dietary diversity and nutritional security, particularly in tropical LMICs [55,56]. Nevertheless, these findings should be interpreted considering key methodological limitations of this review: i) restriction to English publications may introduce language bias, potentially omitting relevant studies from other regions; ii) high heterogeneity among included studies complicates pooled synthesis and may limit generalizability; and iii) some estimates were drawn from prior published syntheses rather than new meta-analyses, which may affect certainty of evidence [7,12,15]. Yet the evidence also illuminates clear opportunities. Deep decarbonization scenarios reveal substantial co-benefits in avoided air-pollution mortality and healthcare expenditure [61,62]. Early-warning systems and urban-heat mitigation measures demonstrate measurable reductions in excess deaths [59]. Such interventions, when equitably financed and contextually adapted, can transform climate action into a primary preventive strategy for public health. Taken together, these thematic insights affirm that safeguarding health under climate change is not solely a technical endeavor; it is an ethical and political imperative requiring cross-sectoral governance, robust financing, and the deliberate centering of equity. The accelerating pace, scale, and complexity of climate-sensitive health impacts leave a narrowing window for decisive action. However, timely, evidence-informed interventions can still redirect trajectories toward resilient, low-carbon, and health-promoting futures.

**Limitations:** this review has several limitations. Substantial heterogeneity in exposure definitions, outcomes, and study designs limits comparability and precision of pooled estimates. Most evidence is observational, introducing potential confounding, bias, and limited causal inference. Exposure misclassification is likely, as many studies rely on ambient rather than individual-level data. Geographic bias persists, with underrepresentation of low- and middle-income countries. Evidence quality varies across domains, particularly for mental health and adaptation effectiveness. Potential publication and language bias cannot be excluded. Finally, complex, non-linear, and compound climate-health interactions are not fully captured, potentially underestimating true risks.

## Conclusion

The evidence assembled here confirms that anthropogenic climate change has already moved from a forecasted health threat to a measurable, present-day determinant of global morbidity, mortality, and economic stability. Across continents and income strata, temperature extremes, altered precipitation patterns, and associated environmental disruptions are amplifying direct physiological stress, reshaping the geography of infectious disease, undermining food and water security, and eroding mental well-being. These impacts converge most acutely where health system capacity, governance, and socioeconomic buffers are weakest, reinforcing profound inequities between and within nations. Notably, this review provides a unique, multi-system synthesis that integrates quantitative evidence across mortality, morbidity, mental health, nutrition, occupational, and pregnancy outcomes, while also linking these health impacts to economic consequences and adaptation interventions [1-7,51-57]. Urgent, coordinated mitigation remains the single greatest public health intervention: trajectories consistent with the Paris Agreement would avert hundreds of thousands of premature deaths annually while generating substantial co-benefits through cleaner air, more resilient food systems, and increased energy security. Yet even the most ambitious decarbonization path cannot eliminate all residual risk. Scalable adaptation-especially heat-health action plans, early warning systems, climate-resilient health facilities, and strengthened surveillance for emerging pathogens-must therefore be financed and implemented in parallel, with particular attention to low- and middle-income countries where gaps are widest. These adaptation priorities are directly informed by the evidence gaps identified in this review, including regional heterogeneity in climate-sensitive outcomes, limited evaluation of intervention efficacy, and incomplete reporting on compound hazards [7,12,15,58,59]. Research priorities correspondingly shift from merely documenting harms to refining actionable solutions. Specifically, this review highlights the need for robust evaluation of adaptation measures, optimization of compound hazard forecasting, and disaggregated assessments capturing gender, age, and occupational vulnerabilities in underrepresented regions [12,26,50,57]. Integrating health metrics into climate economic models will further clarify the true cost of inaction and the value of preventive investment. In sum, safeguarding health on a warming planet is no longer optional or peripheral to climate policy; it is the litmus test of effective climate governance. Rapid, equity-centered mitigation coupled with evidence-based adaptation can still secure a healthier, more sustainable future-but the window for decisive action is closing fast.

### What is known about this topic

- Climate change is a significant threat to human health, with established links to increased mortality from temperature extremes, cardiovascular and respiratory diseases from air pollution, and the changing distribution of vector-borne diseases;
- Specific global estimates, such as millions of annual deaths from non-optimal temperatures and hundreds of thousands from wildfire smoke, have been previously reported by major assessments like the Lancet countdown and the IPCC;
- The economic costs of climate change, including heat-induced labor productivity losses, are recognized as substantial and projected to increase.

### What this study adds

- This review provides a unique, integrated multi-system synthesis that quantitatively links climate exposures not only to mortality and morbidity but also to mental health, nutritional, occupational, and maternal/child outcomes within a single analytical framework;
- It delivers novel pooled estimates for specific outcomes, such as the 5.7% excess in cardiovascular deaths from compound heat-ozone events and the 24% prevalence of climate-related anxiety/depression, offering new granularity for risk assessment;
- The study critically evaluates the evidence for adaptation interventions, directly connecting health burden estimates to the efficacy of solutions like early-warning systems and quantifying the adaptation financing gap (e.g., only 27% of WHO states have fully funded plans).
